# Petchiether A attenuates obstructive nephropathy by suppressing TGF‐β/Smad3 and NF‐κB signalling

**DOI:** 10.1111/jcmm.14454

**Published:** 2019-06-18

**Authors:** Yong‐Ke You, Qi Luo, Wei‐Feng Wu, Jiao‐Jiao Zhang, Hong‐Jian Zhu, Lixing Lao, Hui Y. Lan, Hai‐Yong Chen, Yong‐Xian Cheng

**Affiliations:** ^1^ School of Chinese Medicine, Li Ka Shing Faculty of Medicine The University of Hong Kong Hong Kong China; ^2^ Department of Chinese Medicine The University of Hong Kong‐Shenzhen Hospital Shenzhen China; ^3^ State Key Laboratory of Phytochemistry and Plant Resources in West China Kunming Institute of Botany, Chinese Academy of Sciences Kunming China; ^4^ Guangdong Key Laboratory for Genome Stability & Disease Prevention, School of Pharmaceutical Sciences Shenzhen University Health Science Center Shenzhen China; ^5^ Department of Surgery University of Melbourne Melbourne Australia; ^6^ Department of Medicine and Therapeutics The Chinese University of Hong Kong Hong Kong China; ^7^ Li Ka Shing Institute of Health Sciences The Chinese University of Hong Kong Hong Kong China

**Keywords:** fibrosis, NF‐κB, obstructive nephropathy, petchiether A, TGF‐β/Smad3

## Abstract

Obstructive nephropathy is the end result of a variety of diseases that block drainage from the kidney(s). Transforming growth factor‐β1 (TGF‐β1)/Smad3‐driven renal fibrosis is the common pathogenesis of obstructive nephropathy. In this study, we identified petchiether A (petA), a novel small‐molecule meroterpenoid from *Ganoderma*, as a potential inhibitor of TGF‐β1‐induced Smad3 phosphorylation. The obstructive nephropathy was induced by unilateral ureteral obstruction (UUO) in mice. Mice received an intraperitoneal injection of petA/vehicle before and after UUO or sham operation. An in vivo study revealed that petA protected against renal inflammation and fibrosis by reducing the infiltration of macrophages, inhibiting the expression of proinflammatory cytokines (interleukin‐1β and tumour necrosis factor‐α) and reducing extracellular matrix deposition (α‐smooth muscle actin, collagen I and fibronectin) in the obstructed kidney of UUO mice; these changes were associated with suppression of Smad3 and NF‐κB p65 phosphorylation. Petchiether A inhibited Smad3 phosphorylation in vitro and down‐regulated the expression of the fibrotic marker collagen I in TGF‐β1‐treated renal epithelial cells. Further, we found that petA dose‐dependently suppressed Smad3‐responsive promoter activity, indicating that petA inhibits gene expression downstream of the TGF‐β/Smad3 signalling pathway. In conclusion, our findings suggest that petA protects against renal inflammation and fibrosis by selectively inhibiting TGF‐β/Smad3 signalling.

## 1 INTRODUCTION

Renal fibrosis is the most common progressive process in the pathogenesis of chronic kidney diseases, degrading kidney function and eventually causing end‐stage renal disease in patients.^1^, ^2^, ^3^, ^4^, ^5^ Renal fibrosis is characterised by extracellular matrix deposition in glomerular and tubulointerstitial tissue. Increasing evidence shows that obstructive nephropathy, which is commonly caused by urolithiasis, benign prostatic hyperplasia and pelvic or ureteral tumours, leads to proximal tubular cell loss and interstitial fibrosis. The unilateral ureteral obstruction (UUO) model is widely used to study the mechanisms of tubulointerstitial fibrosis via surgically induced obstructive renal injury.^6^ In experimental animal models and patients with obstructive nephropathy, the major pathological features in local are infiltration of inflammatory cells, secretion of proinflammatory cytokines, such as interleukin‐1β (IL‐1β), tumour necrosis factor‐α (TNF‐α) and monocyte chemoattractant protein‐1 (MCP‐1) and the accumulation of fibrotic markers, such as collagen I, fibronectin and α‐smooth muscle actin (α‐SMA). The TGF‐β/Smad signalling pathway may be a viable therapeutic target for treating renal fibrosis.^7^, ^8^, ^9^ Treatments that manipulate TGF‐β/Smad signalling have shown beneficial effects in the kidneys of laboratory animals.^10^, ^11^, ^12^, ^13^, ^14^, ^15^, ^16^ However, this treatment is not available in current clinical practice because of several associated problems, such as safety issues and immunological tolerance.

The genus *Ganoderma* (also called Lingzhi) is a medicinal fungal genus that includes various species, for example, *G petchii*, *G australe* and *G lucidum*. *Ganoderma* is traditionally used in China to promote health and longevity, lower the risks of cancer and heart disease, protect against liver and kidney diseases, and boost the immune system.^17^, ^18^, ^19^, ^20^, ^21^ Derivatives of *Ganoderma* have shown a renoprotective effect in diabetic nephropathy, chronic glomerulonephritis and tubulointerstitial fibrosis.^22^, ^23^, ^24^, ^25^

Petchiether A (petA), a novel small‐molecule meroterpenoid isolated from the fruiting body of *G petchii*, inhibits fibronectin in rat kidney tubular epithelial cells.^26^ However, the in vivo mechanisms and functional significance of this activity remain unknown. The present study examined the effects of petA on UUO‐induced obstructive kidney injury and its underlying mechanisms both in vivo and in vitro.

## 2 MATERIALS AND METHODS

### 2.1 Drug isolation and identification

The fruiting bodies of *G petchii* and *G australe* were purchased from a market selling Chinese medical materials in Zhonghao‐Luoshi‐Wan, Kunming, Yunnan Province, China. The material was identified by Prof. Zhu‐Liang Yang at the Kunming Institute of Botany, Chinese Academy of Sciences.

**Figure 1:**
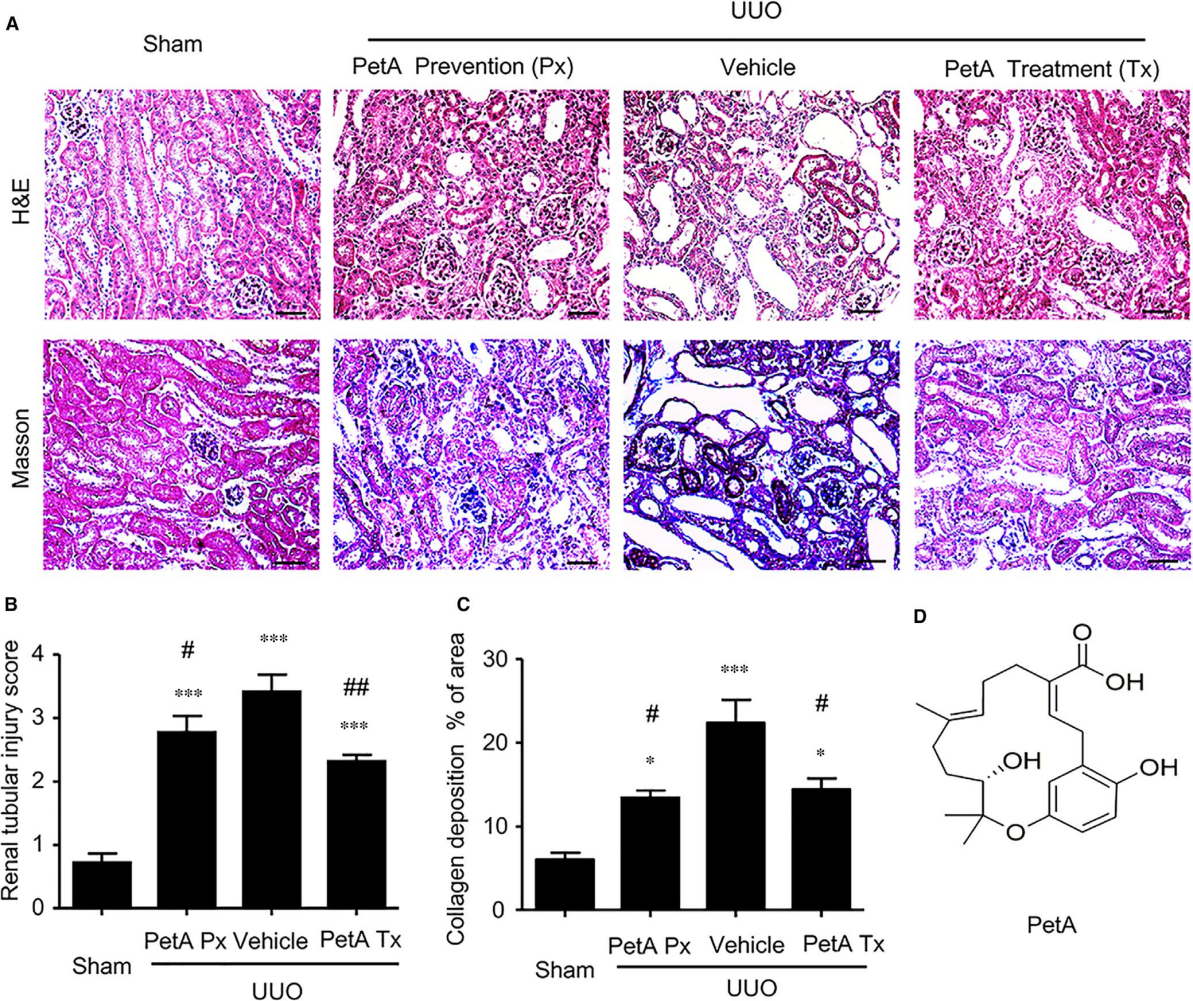
Petchiether A (PetA) attenuates the collagen deposition and histological injury observed in the obstructed kidneys at 5 d after unilateral obstructive (UUO) operation. Mice receiving daily intraperitoneal injection of vehicle or petA (40 mg/kg/d) 4 d before or right after UUO operation were killed 5 d after UUO. A, Haematoxylin and eosin and Masson's trichrome staining. B, Semi‐quantitative analysis of the interstitial injury score and (C) relative collagen deposition area of the obstructive kidney in each group. D, The structure of petA. Data represent the mean ± SEM for 6‐8 mice per group. *P < 0.05, ***P < 0.001 vs the sham group; ^#^ *P* < 0.05, ^##^ *P* < 0.01 vs the vehicle UUO group. Bar = 50 μmol/L. Magnification ×200. PetA Px, PetA prevention; PetA Tx, PetA treatment

The procedure of PetA isolation was previously described.^26^ The powders of fruiting bodies of *G petchii* were extracted by reflux with 70% ethyl alcohol (EtOH). The extraction was suspended in water, followed by the participation with ethyl acetate (EtOAc). Eight parts (Fr1‐Fr8) were separated from the EtOAc extract using a MCI gel CHP 20P column (75‐150 μm) washing with gradient aqueous methyl alcohol (MeOH) from 10%‐100%. The Fr5 was further separated into seven portions (Fr5.1‐Fr5.7) using a MCI gel CHP 20P column eluting with gradient aqueous MeOH (20%‐100%). Among the fragments, pet A was separated from fr5.6 using Sephadex LH‐20 (MeOH) followed by an RP‐18 column (MeOH/H_2_O, 30:70‐100:0), and preparative TLC (CHCl_3_/Me_2_CO, 8:1). Because of the low content of petA in *G petchii*, the compound was also enriched from *G australe* (176 mg from 90 kg fungus) using a similar isolation procedure and structurally identified using multiple spectroscopic methods and further confirmed by the Mosher's method (Figure 1D). The purity of petA was over 98%.

### 2.2 Animal model

Ten‐ to 12‐week‐old male C57BL/6J mice (bodyweight 20‐25 g) were used for this study. Unilateral ureteral obstruction was performed using an established protocol as described previously.^27^, ^28^ To evaluate the effect of petA on renal fibrosis, we randomised the mice into four groups (n = 5‐8 per group): (a) the sham‐operated group; (b) the UUO group, in which mice received intraperitoneal (ip) injections of vehicle for four consecutive days; (c) the prevention group, in which mice received 4 consecutive days of petA (ip) before UUO; and (d) the treatment group, in which mice received 4 days of petA (ip) after UUO. Petchiether A was dissolved in dimethyl sulfoxide (DMSO). All mice were killed on day 5 after left ureter ligation. Kidney tissues were collected for histology, immunohistochemistry, Western blotting and real‐time reverse transcript‐PCR analysis as previously described.^29^ All studies were approved by the Animal Experimentation Ethics Committee of the University of Hong Kong, and the experimental methods were performed in accordance with the approved guidelines.

### 2.3 Morphological and immunohistochemical analysis

To examine the changes in renal morphology, we stained formalin‐fixed, paraffin‐embedded sections (3 µm) with haematoxylin and eosin or a Masson's trichrome staining kit (ScyTek Laboratories, West Logan, UT) according to the manufacturer's instructions and as previously described.^29^, ^30^ Immunohistochemistry was performed in paraffin sections using a microwave‐based antigen retrieval method.^31^ The primary antibodies used in this study included antibodies against TNF‐α, IL‐1β, TGF‐β1, fibronectin (Santa Cruz Biotechnology, Santa Cruz, CA), F4/80 (AbD Serotec, Kidlington, UK), phospho‐Smad3 (Rockland Immuno‐chemicals, Gilbertsville, PA), α‐SMA (Sigma, St. Louis, MO), collagen I (Southern Biotech, Birmingham, AL) and phospho‐nuclear factor κ light chain enhancer of activated B cells (NF‐κB)/p65 (Abcam, Cambridge, MA). Positive signals were analysed with the quantitative Image Analysis System (Image‐Pro Plus 7.0; Media Cybernetics, Bethesda, MD) as described previously.^32^, ^33^

### 2.4 Cell culture

Human kidney (proximal tubular epithelial) cell line 2 (HK‐2) cells, a normal adult human renal tubular epithelial cell line, were cultured in serum‐free DMEM/Ham's F12 medium (Invitrogen Life Technologies, Gaithersburg, MD). To investigate the effect of petA on TGF‐β1‐induced phosphorylation of Smad3, we pre‐treated HK‐2 cells with the indicated doses of petA for 12 hours before adding TGF‐β1 (2.5 ng/mL) for another 12 hours. To determine the preventive and therapeutic effects of petA on TGF‐β1/Smad3 signalling‐induced collagen I expression, we incubated HK‐2 cells with petA (25 μmol/L) for 12 hours before or after 12 hours of TGF‐β1 (2.5 ng/mL). Each experiment was repeated independently at least three times.

### 2.5 MTT assay

HK‐2 cells were seeded into 96‐well plates at a density of 1 × 10^5^ cells/mL in a volume of 200 μL per well and allowed to attach for 24 hours. The cells were starved with serum‐free medium for 24 hours and then incubated with the indicated amounts of petA (1, 2.5, 5, 10, 25, 50, 100 μmol/L) for 12 hours. Cell viability was determined by adding 20 μL of the reagent 3‐[4,5‐dimethylthiazol‐2‐yl‐]‐2,5‐diphenyltetrazolium bromide (MTT) at a concentration of 5 mg/mL and incubating the cells for 4 hours at 37°C. The MTT‐containing medium was removed, and 150 μL of DMSO was added to dissolve the formazan crystals. The absorbance value was measured at 540 nm using a microplate reader (Bio‐Tek Instruments, Inc, Ontario, Canada).

### 2.6 **Smad3**‐**responsive promoter assay**

HK‐2 cells were transiently transfected with the Smad3‐responsive promoter p(CAGA)12‐Luc (kindly provided by Professor Hong‐Jian Zhu, University of Melbourne) as described previously.^28^ The PGL3 Basic plasmid was co‐transfected into the cells as a control. After transfection, cells were treated with petA (5, 25, 50 μmol/L) for 12 hours, followed by the addition of TGF‐β1 (2.5 ng/mL) for another 12 hours. The luciferase activity of p(CAGA)12 was analysed using a Promega Luciferase Assay kit (Promega Corporation, Wisconsin, USA) according to the manufacturer's instructions and measured using a PerkinElmer 2030 Multilabel Luminescence Microplate Reader (PerkinElmer Life and Analytical Sciences, Finland). Three independent experiments were performed.

### 2.7 Western blot analysis

Protein was extracted from the kidney tissues and cultured HK‐2 cells using radio‐immunoprecipitation assay lysis buffer, and Western blot analysis was performed as described previously.^28^ Five percent bovine serum albumin (BSA) was used to block non‐specific binding before the membranes were incubated with primary antibody overnight at 4°C. The antibodies used in this study included primary antibodies against collagen I (Southern Biotech), fibronectin (Santa Cruz Biotechnology), phospho‐NF‐κB/p65, NF‐κB/p65, phospho‐Smad3 and Smad3 (Cell Signalling Technology Inc, Danvers, MA) and secondary antibodies labelled with LI‐COR IRDye 800 (Rockland Immuno‐chemicals). Signal detection was performed using the Odyssey infrared imaging system (LI‐COR Biosciences, Lincoln, NE) and quantified by Imagej software (National Institutes of Health). The expression level of each protein was normalised to the expression level of β‐actin and is expressed as the mean ± SEM

### 2.8 RNA extraction, quantitative real‐time PCR

Total RNA was isolated from the renal tissues and cultured HK‐2 cells using TRIzol reagent from Invitrogen according to the manufacturer's instructions, and real‐time PCR was performed using Bio‐Rad IQ SYBR Green Supermix with Option 2 (Bio‐Rad, Hercules, CA) as previously described.^27^ The primers used in this study for mouse IL‐1β, TNF‐a, TGF‐β1, a‐SMA, collagen I and fibronectin mRNAs have been described previously.^27^, ^28^, ^30^, ^31^, ^32^, ^34^ The housekeeping gene β‐actin was used as an internal control. The expression level of each mRNA of interest was normalised to that of β‐actin and expressed as the mean ± SEM

### 2.9 Statistical analysis

All the data obtained from this study are expressed as the mean ± SEM from at least three independent experiments or groups of five to eight mice each. Statistical analyses were performed with one‐way ANOVA followed by the Newman‐Keuls post hoc test. The tests were performed in GraphPad Prism 5 (GraphPad Software, La Jolla, CA). A *P*‐value <0.05 was considered significant.

## 3 RESULTS

### 3.1 Toxicity of petA

To detect the toxicity of petA, we incubated human proximal tubular cells (HK‐2) with the indicated doses of petA. Cell viability was detected by MTT (3‐[4,5‐dimethylthiazol‐2‐yl‐]‐2,5‐diphenyltetrazolium bromide) experiments. Petchiether A did not affect the viability or proliferation of the cells at any of the tested doses (1, 2.5, 5, 10, 25, 50, 100 μmol/L; Figure S1).

### 3.2 PetA attenuates kidney injury after UUO

We evaluated the effect of petA on renal fibrosis in a typical model of renal interstitial fibrosis caused by UUO. In the preliminary study, based on our previous study of *G lucidum*,^35^ petA was administered immediately after UUO operation by ip injection at dosages of 20 and 40 mg/kg/d (n = 8 in the 20 mg/kg group, n = 5 in the 40 mg/kg group). Mice were killed on day 5 after UUO. Morphological and Western blot analyses showed that 40 mg/kg petA significantly decreased histological injury and Smad3 phosphorylation and collagen I expression (Figure S2). Therefore, 40 mg/kg of petA was used in the following prevention and treatment studies.

The haematoxylin and eosin and Masson's trichrome staining analyses indicated the obstructed kidney showed severe tubulointerstitial damage, such as tubular dilatation, atrophy, infiltration of inflammatory cells and accumulation of collagen deposition compared to the sham‐operated kidney (Figure 1A). However, administration of petA (40 mg/kg), initiated either immediately after or 4 days before the UUO procedure, markedly reduced these changes compared with those in the vehicle group (Figure 1A); this result was further confirmed by semi‐quantification of the tubulointerstitial damage observed in haematoxylin and eosin‐ and Masson's trichrome‐stained sections of the kidney tissue (Figure 1B,C). Taken together, these results indicate that the use of petA for the prevention and treatment of renal fibrosis in mice protects against renal histological damage and fibrosis.

### 3.3 PetA ameliorates renal inflammation and fibrosis in the kidney after UUO

**Figure 2:**
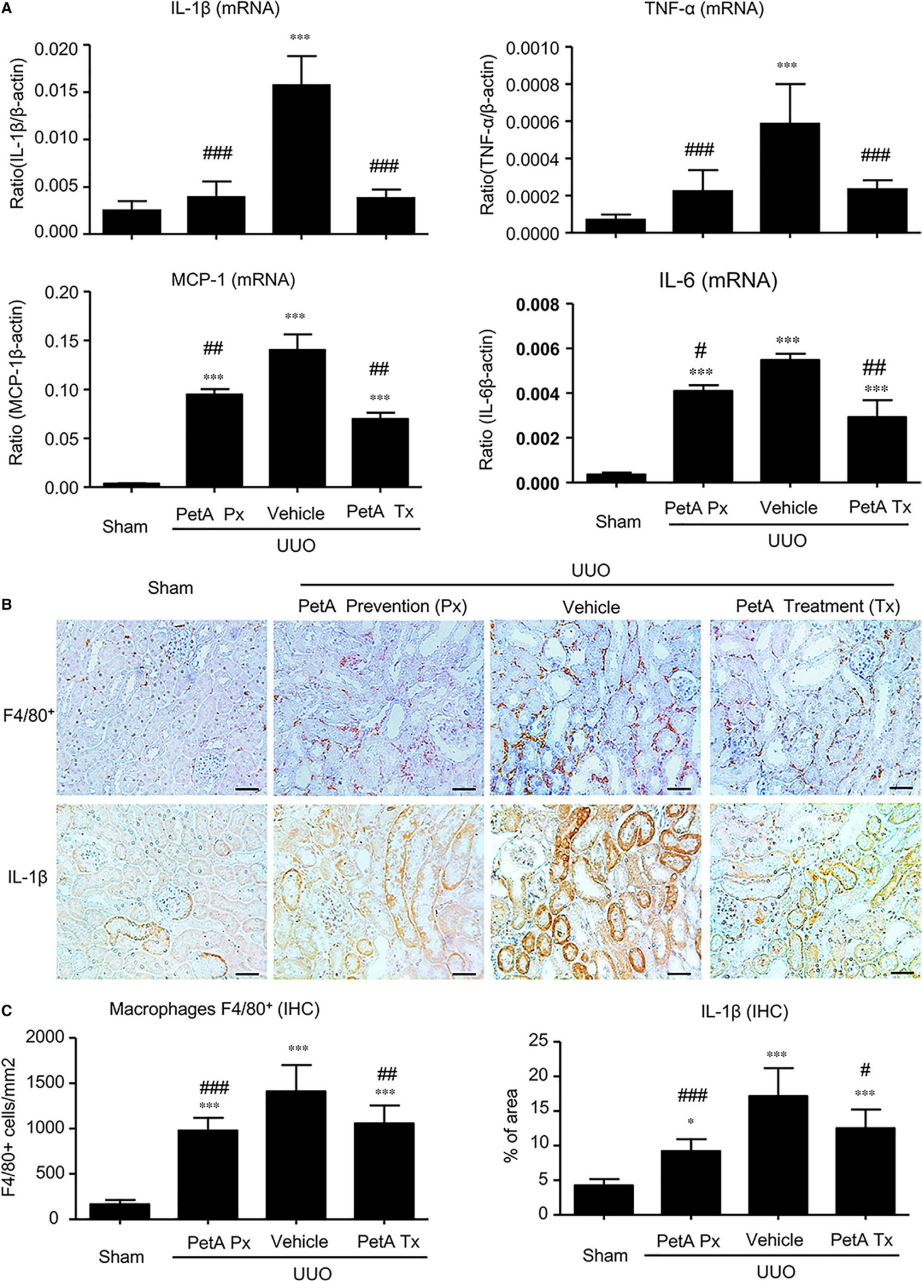
Petchiether A (PetA) inhibits the expression of proinflammatory cytokines and infiltration of macrophages in the obstructed kidneys of unilateral obstructive (UUO) mice. Mice receiving daily intraperitoneal injection of vehicle or petA (40 mg/kg/d) 4 d before or right after UUO operation were killed 5 d after UUO. A, interleukin‐1β (IL‐1β), tumour necrosis factor‐α (TNF‐α), MCP‐1 and IL‐6 expression were examined by quantitative real‐time PCR, as indicated. B, Immunohistochemical staining and (C) semi‐quantitative analysis of F4/80^+^ and IL‐1β expression. Data represent the mean ± SEM for 6‐8 mice per group. *P < 0.05, ***P < 0.001 vs the sham group; ^#^ *P* < 0.05, ^##^ *P* < 0.01, ^###^ *P* < 0.001 vs the vehicle UUO group. Bar = 50 μmol/L. Magnification ×200. PetA Px, PetA prevention; PetA Tx, PetA treatment

**Figure 3:**
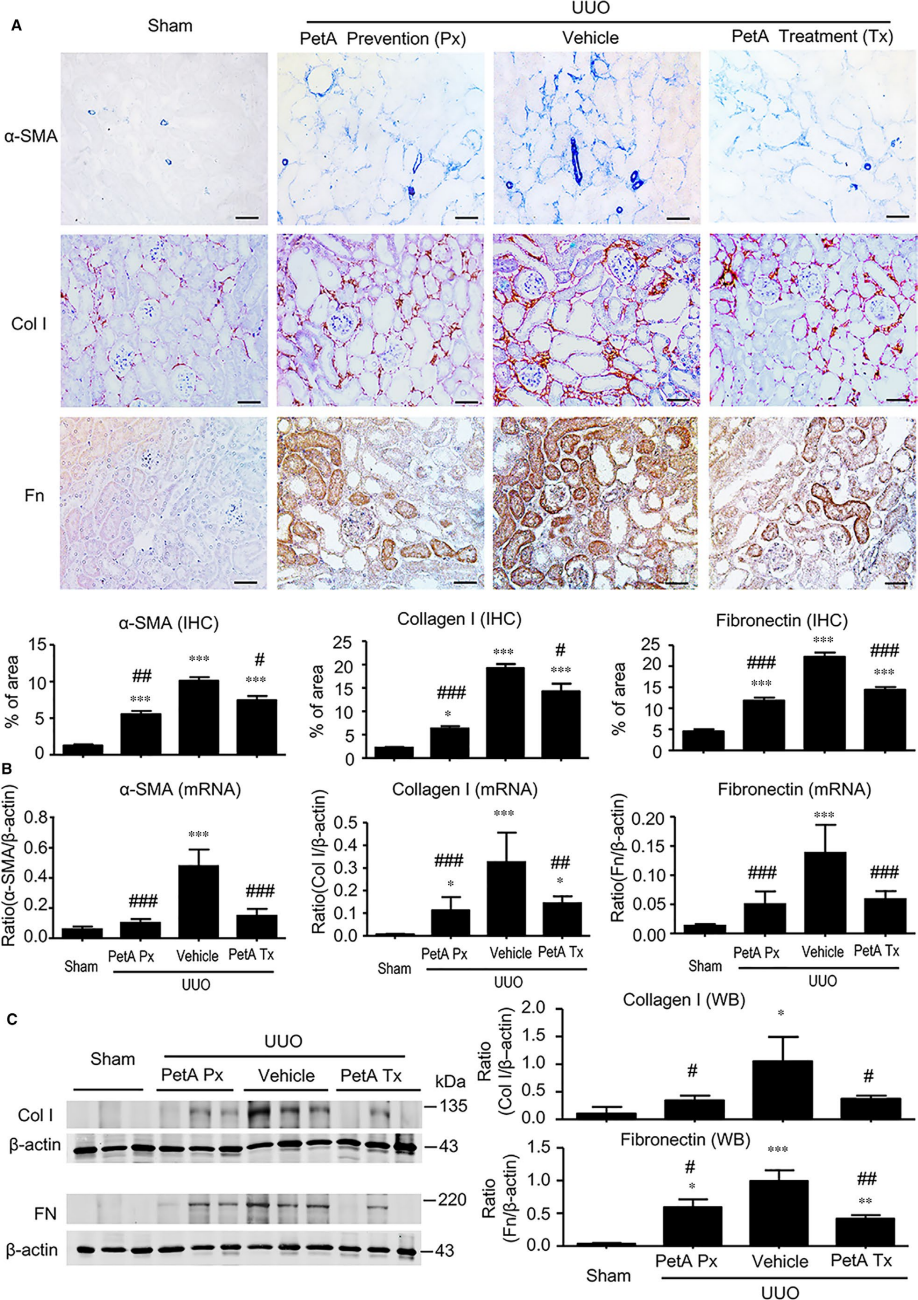
Petchiether A (PetA) inhibits the expression of fibronectin, collagen 1 and α‐smooth muscle actin (α‐SMA) in the obstructed kidneys at 5 d after unilateral obstructive (UUO) operation. Mice receiving daily intraperitoneal injection of vehicle or petA (40 mg/kg/d) 4 d before or right after UUO operation were killed 5 d after UUO. A, Immunohistochemical staining and quantitative analysis for the expression of α‐SMA, collagen 1 and fibronectin. B, Quantitative real‐time PCR analysis of α‐SMA, collagen 1 and fibronectin mRNA expression. C, Western blot and semiquantitative analysis of collagen 1 and fibronectin, respectively. Data represent the mean ± SEM for 6‐8 mice per group. *P < 0.05, **P < 0.01, ***P < 0.001 vs the sham group; ^#^ *P* < 0.05, ^##^ *P* < 0.01, ^###^ *P* < 0.001 vs the vehicle UUO group. Bar = 50 μmol/L. Magnification ×200. PetA Px, PetA prevention; PetA Tx, PetA treatment

We then examined the effect of petA on renal inflammation and fibrosis in UUO mice. Immunohistochemistry and real‐time PCR analysis revealed that, compared with the sham‐operated mice, UUO vehicle mice developed moderate renal inflammation including a marked up‐regulation of proinflammatory cytokines/chemokines (TNF‐α, IL‐1β, MCP‐1 and IL‐6) and renal infiltration of F4/80^+^ macrophages (Figure 2). However, all these inflammatory features were significantly decreased in the obstructed kidney after 5 days of UUO in the petA (40 mg/kg) prevention and petA (40 mg/kg) treatment group mice (Figure 2). Further studies also revealed that moderate renal fibrosis, as indicated by the expression of α‐SMA, collagen I and fibronectin mRNA and the accumulation of the corresponding matrix proteins, occurred in UUO vehicle mice but was substantially attenuated in mice that received petA (40 mg/kg) 4 days before or immediately after UUO operation (Figure 3).

### 3.4 PetA ameliorates renal inflammation and fibrosis in UUO mice and is associated with the suppression of the NF‐κB and TGF‐β1/Smad3 signalling pathways

**Figure 4:**
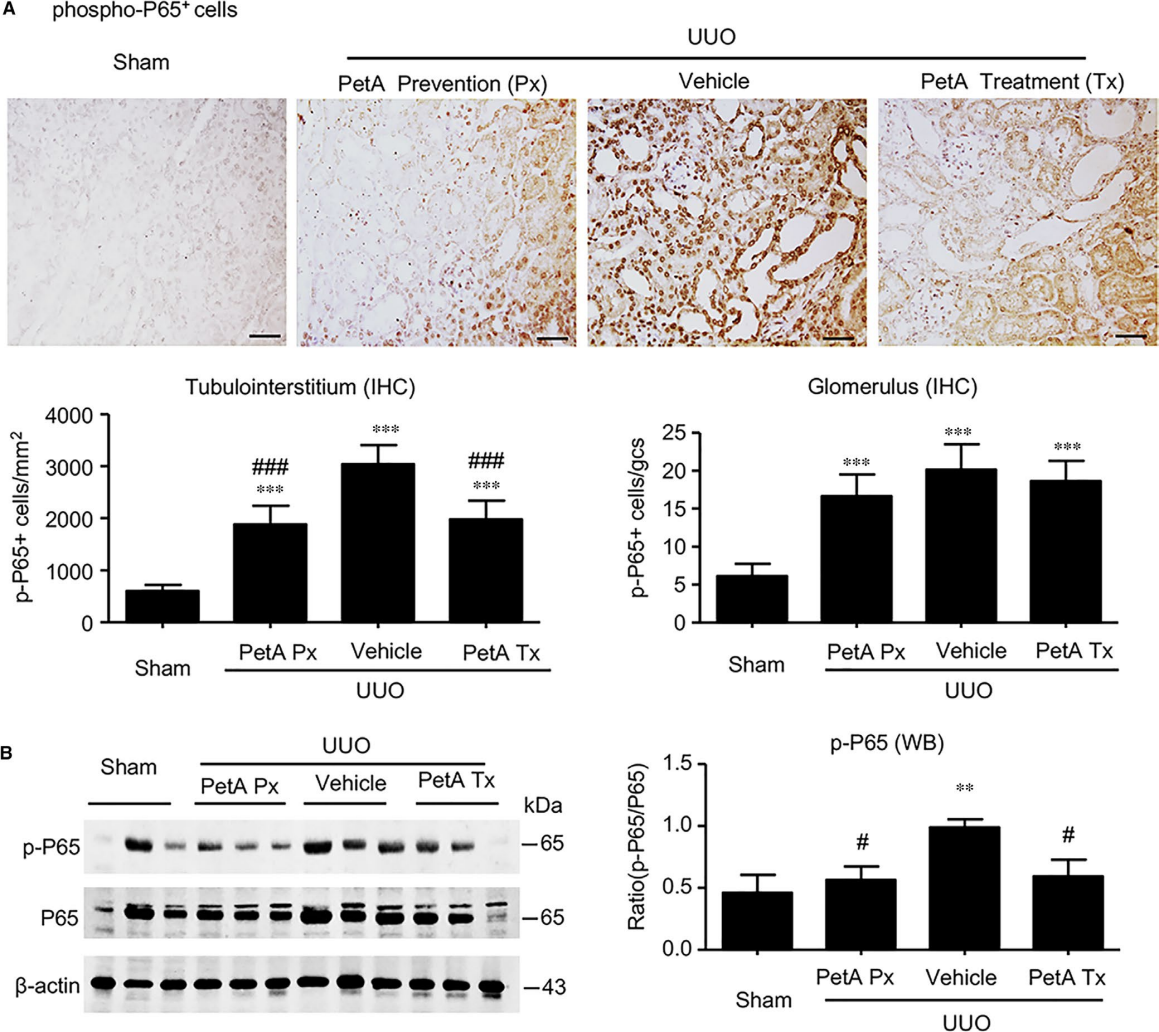
Petchiether A (PetA) inhibits NF‐κB signalling in the obstructed kidneys of unilateral obstructive (UUO) mice. Mice receiving daily intraperitoneal injection of vehicle or petA (40 mg/kg/d) 4 d before or right after UUO operation were killed 5 d after UUO. A, Immunohistochemical staining and quantitative analysis of nuclear phospho‐NF‐κB/p65 (p‐p65). B, Western blot and quantitative analysis of phospho‐NF‐κB/p65 (p‐p65) protein expression. Data represent the mean ± SEM for 6‐8 mice per group. **P < 0.01, ***P < 0.001 vs the sham group; ^#^ *P* < 0.05, ^###^ *P* < 0.001 vs the vehicle UUO group. Bar = 50 μmol/L. Magnification ×200. PetA Px, PetA prevention; PetA Tx, PetA treatment

We next investigated the underlying signalling mechanisms by which petA protects against obstructive kidney injury. First, we examined the NF‐κB inflammation signalling pathway. Immunohistochemistry and Western blot analysis revealed significantly elevated concentrations and nuclear translocation of phosphorylated p65 subunit in the obstructed kidneys of the vehicle UUO group, and these measures were markedly decreased in the obstructive kidneys of the petA (40 mg/kg) prevention and petA (40 mg/kg) treatment group mice (Figure 4), suggesting that petA may protect against renal inflammation during UUO via NF‐κB signalling.

**Figure 5:**
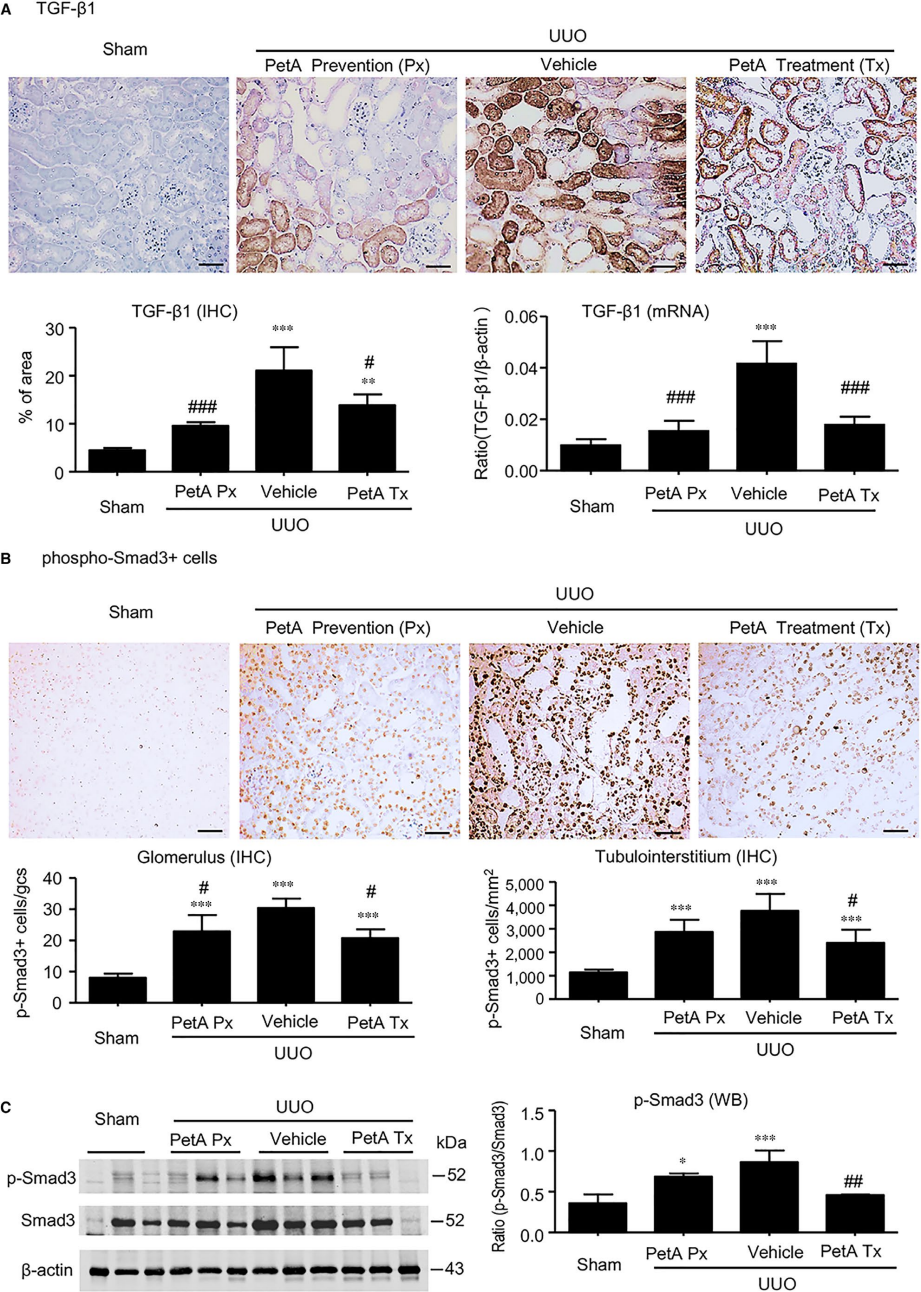
Petchiether A (PetA) inhibits transforming growth factor‐β1 (TGF‐β1)/Smad3 signalling in the obstructive kidneys of unilateral obstructive (UUO) mice. Mice receiving daily intraperitoneal injection of vehicle or petA (40 mg/kg/d) 4 d before or right after UUO operation were killed 5 d after UUO. A, TGF‐β1 expression was examined by immunohistochemistry and quantitative real‐time PCR. Phosphorylation of Smad3 was examined by immunohistochemistry (B) and quantitative Western blot analysis (C). Data represent the mean ± SEM for 6‐8 mice per group. *P < 0.05, **P < 0.01, ***P < 0.001 vs the sham group; ^#^ *P* < 0.05, ^##^ *P* < 0.01, ^###^ *P* < 0.001 vs the vehicle UUO group. Magnification ×200. PetA Px, PetA prevention; PetA Tx, PetA treatment

Furthermore, we also found a significant up‐regulation of renal TGF‐β1 at the mRNA and protein levels (Figure 5A) in UUO vehicle mice, and this up‐regulation was associated with enhanced Smad3 signalling, as detected by increased concentrations and nuclear localisation of phosphorylated Smad3 in glomerular and tubulointerstitial cells (Figure 5B,C). However, these increases were abolished by petA (40 mg/kg) as prevention or treatment in UUO mice (Figure 5). These findings suggest that petA may protect against renal fibrosis in UUO via the TGF‐β/Smad3 signalling pathway.

### 3.5 PetA attenuates fibrosis by inhibiting phosphorylation of Smad3

**Figure 6:**
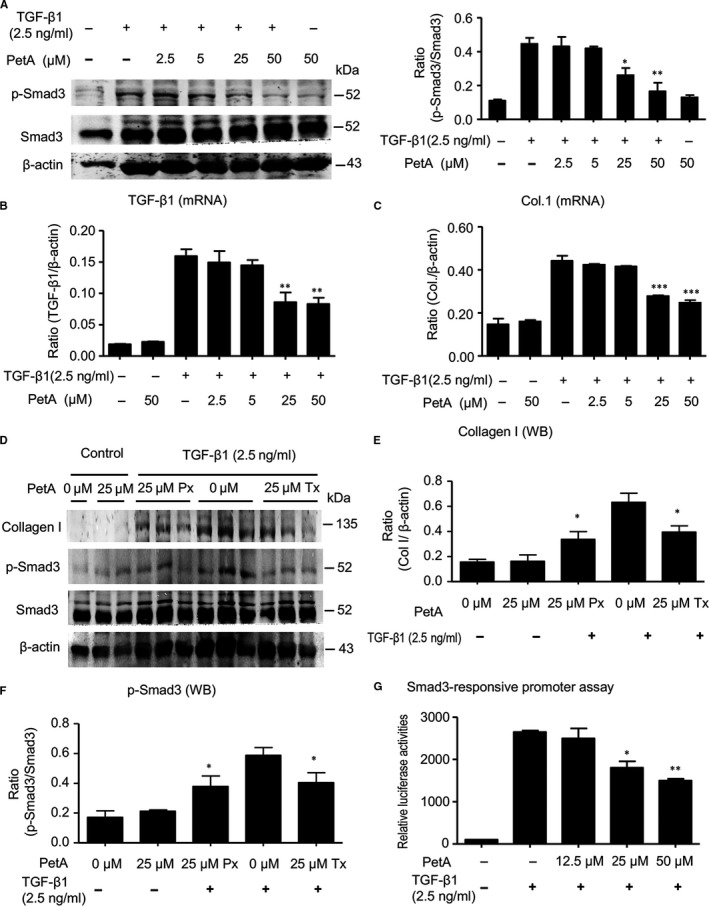
Petchiether A (PetA) attenuates collagen I expression by inhibiting transforming growth factor‐β1 (TGF‐β1)‐induced phosphorylation of Smad3 in cultured HK‐2 cells. A, HK‐2 cells were pre‐treated with the indicated dose of petA (2.5, 5, 25, 50 μmol/L) or vehicle for 12 h before stimulation with TGF‐β1 (2.5 ng/mL) for another 12 h and examined for Smad3 phosphorylation by Western blotting. Representative Western blots and quantitative analysis of Smad3 phosphorylation are shown. TGF‐β1 mRNA expression (B) and (C) collagen 1 mRNA expression were measured using quantitative real‐time PCR. D, HK‐2 cells were pre‐treated with petA (25 μmol/L) or vehicle for 12 h before (Px group) or after (Tx group) stimulation with TGF‐β1 (2.5 ng/mL) for another 12 h. Representative Western blots show that the addition of petA (25 μmol/L) can inhibit TGF‐β1‐induced up‐regulation of collagen I expression (E) and Smad3 phosphorylation (F). G, PetA inhibits Smad3‐specific luciferase reporter p(CAGA)‐luc in HK‐2 cells. HK‐2 cells were cotransfected with the p(CAGA)‐luc plasmid, followed by pre‐treatment with the indicated dose of petA (12.5, 25, 50 μmol/L) or vehicle for 4 h before stimulation with TGF‐β1 (2.5 ng/mL) for another 12 h. Luciferase activity in the control group was set at 100%. Compared with the increased p(CAGA)‐luc reporter activity in petA‐untreated HK‐2 cells under TGF‐β1 stimulation, petA at a dose of 25 μmol/L significantly inhibited (CAGA) reporter activity. Data represent the mean ± SEM for at least three independent experiments. *P < 0.05, **P < 0.01 compared with petA‐untreated cells under TGF‐β1 stimulation. PetA Px, PetA prevention; PetA Tx, PetA treatment

To examine whether petA protected against fibrosis by suppressing TGF‐β1‐induced Smad3 phosphorylation, HK‐2 tubular epithelial cells were treated with TGF‐β1 with/without petA. Western blot results demonstrated that petA significantly inhibited TGF‐β1‐induced Smad3 phosphorylation in a dose‐dependent manner (Figure 6A), and real‐time PCR analysis revealed a dramatic decrease in endogenous TGF‐β1 and collagen 1 expression (Figure 6B,C). However, the baseline level of endogenous TGF‐β1 in PetA treated HK‐2 cells had no significant change. Further, HK‐2 cells were pre‐treated with or without petA (25 μmol/L) for 12 hours either before (prevention group) or immediately after (treatment group) stimulation with TGF‐β1 (2.5 ng/mL). Western blot analysis showed that the addition of petA (25 μmol/L) inhibited the phosphorylation of Smad3 and the up‐regulated expression of the fibrotic marker collagen I by TGF‐β1 (Figure 6D‐F). The Smad3‐responsive promoter assay results demonstrated that petA dose‐dependently suppressed TGF‐β1‐induced Smad3‐responsive promoter activity, indicating that petA significantly inhibited gene expression downstream of the TGF‐β/Smad3 signalling pathway (Figure 6G).

## 4 DISCUSSION

We report for the first time that petA, a novel small molecule extracted from the traditional Chinese medicine *Ganoderma*, markedly attenuates renal inflammation and fibrosis in UUO by inhibiting NF‐κB and TGF‐β1/Smad3 signalling. Petchiether A can inhibit TGF‐β1‐induced Smad3 phosphorylation, suggesting a potential role of petA as an effective agent against renal fibrogenesis.

The antifibrotic actions of petA may involve multiple mechanisms. Because TGF‐β1/Smad3 signalling is the critical pathway of renal fibrosis,^3^, ^4^, ^36^, ^37^, ^38^ we first examined the effect of petA on the activation of TGF‐β1/Smad3 signalling in the UUO model. Our results indicated that administering petA to prevent or treat UUO suppressed TGF‐β1 expression and blocked the phosphorylation of Smad3, subsequently attenuating the expression of fibrotic genes (including α‐SMA, collagen I and fibronectin). In support of this conclusion, we demonstrated that petA blocked TGF‐β1‐induced phosphorylation of Smad3 and expression of collagen I in cultured HK‐2 cells. These results are consistent with our previous report that the inhibitory effects of PetA on fibronectin production in rat kidney tubular epithelial cells (NRK52E) under the stimulation of TGF‐β1.^26^ Therefore, the inhibition of renal fibrosis by petA is mediated at least partly by the suppression of the TGF‐β1/Smad axis in the obstructed kidneys.

We previously demonstrated that Smad3 is a key Smad protein that mediates fibrosis in multiple organs and tissues.^3^, ^28^ Inhibition of Smad3 using specific inhibitors reduces fibrosis.^39^, ^40^, ^41^, ^42^ In this study, we found that petA is a potential inhibitor of TGF‐β/Smad3 activation, which suggests that petA may relieve renal fibrosis as well as cirrhosis, cardiac fibrosis and lung fibrosis.

Inflammation plays a central mechanism in the initiation and maintenance of kidney injury, and a suppressed inflammatory response reduces the extent of renal fibrosis.^43^, ^44^ Our results indicate that administering petA to prevent or treat UUO can suppress the expression of multiple proinflammatory cytokines, such as TNF‐α, IL‐β, MCP‐1 and IL‐6, and inhibit macrophage infiltration, suggesting that inhibition of the inflammatory response is also one of the mechanisms by which petA ameliorates renal fibrosis.

Furthermore, several mechanisms may contribute to the anti‐inflammatory effects of petA. We previously found that the activation of TGF‐β/Smad3 is associated with a decreased expression of Smad7, an inhibitory Smad, in UUO, hypertensive and diabetic kidneys.^27^, ^45^, ^46^ The decrease in Smad7 enhances NF‐κB P65 phosphorylation, which consequently aggravates inflammation in the kidneys.^27^, ^45^, ^46^ Another possible mechanism may be the overexpression of TGF‐β1 in UUO kidneys (Figure 5A). Findings from other research teams indicate that TGF‐β1 activates renal tubular cells and immune system cells to produce inflammatory cytokines and further promote the inflammatory response, which, in turn, amplifies fibrosis and tubular injury.^47^, ^48^, ^49^ The mechanisms by which petA attenuates inflammation will be explored in future studies.

In conclusion, our findings suggest that petA is a potential natural small‐molecule inhibitor of TGF‐β1‐induced Smad3 phosphorylation. The beneficial effect of petA may occur through the inhibition of TGF‐β1/Smad3 and NF‐κB signalling, subsequently reducing renal fibrosis and inflammation in UUO kidneys. Our studies may provide a useful natural therapeutic agent for the prevention and treatment of renal fibrosis during the progression of fibrotic kidney disease.

## CONFLICT OF INTEREST

The authors have declared that they have no conflicts of interest.

## AUTHOR CONTRIBUTION

HYC designed and supervised the study, interpreted the data and wrote the manuscript. YKY and WFW performed the experiments, analysed the data and drafted the manuscript. HYL, ZJZ and LXL were involved in the conception of experiments and data interpretation. YXC designed the petA extraction and identification protocols; QL and JJZ performed the isolation and purification of petA. All authors participated in reviewing and editing subsequent drafts of the manuscript and approved the final content of the manuscript.

## Data Availability

The datasets generated during and/or analysed during the current study are available from the corresponding author on reasonable request.
