# Supplementary material for: Petchiether A attenuates obstructive nephropathy by suppressing TGF‐β/Smad3 and NF‐κB signalling
Source: J Cell Mol Med. 2019 Jun 18;23(8):5576–87. doi: 10.1111/jcmm.14454 (PMC6652659; doi:10.1111/jcmm.14454)
Supplement: Supplementary file 1 [file JCMM-23-5576-s001.pdf]

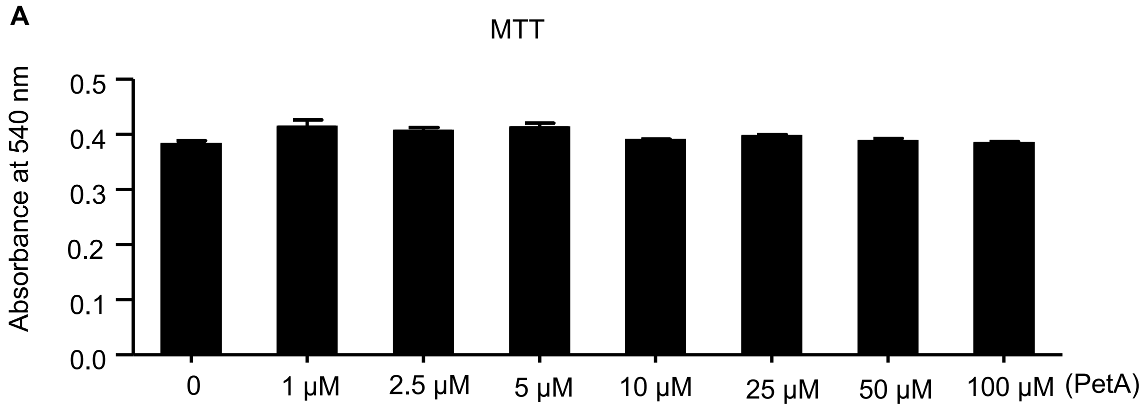

Supplement Figure S1. PetA does not affect the viability or proliferation of HK-2 cells. HK-2 cells were incubated with different concentrations of petA for 12 hours. Cell viability was determined by the MTT assay.

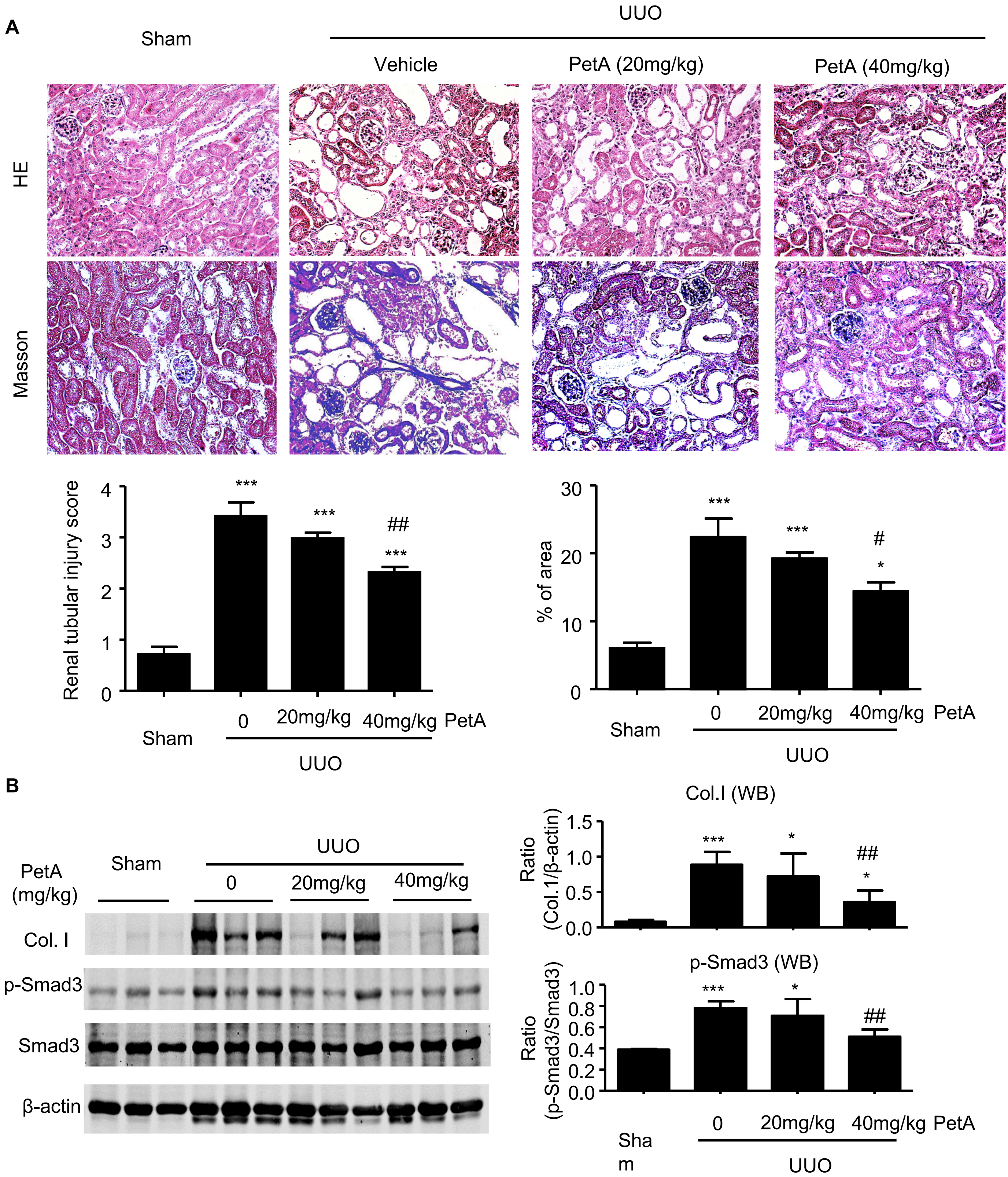

Supplement Figure S2. PetA ameliorates renal interstitial fibrosis in UUO kidneys. PetA was intraperitoneal injected at the dosage of 0, 20 and 40 mg/kg per day right after UUO. Mice were sacrificed at day 5 after UUO. (A) HE & Masson staining, and (B) western blot analyses demonstrated that 40mg/kg of PetA per day significantly decrease phosphorylation of Smad3 and the expression of collagen I. * p<0.05, ***p<0.001 vs sham group; # p<0.05, ##p<0.01 vs UUO + vehicle , n=5 in each group. Magnification 200×.
